# Quantum secret sharing via local operations and classical communication

**DOI:** 10.1038/srep16967

**Published:** 2015-11-20

**Authors:** Ying-Hui Yang, Fei Gao, Xia Wu, Su-Juan Qin, Hui-Juan Zuo, Qiao-Yan Wen

**Affiliations:** 1 State Key Laboratory of Networking and Switching Technology, Beijing University of Posts and Telecommu-nications, Beijing, 100876, China; 2 School of Mathematics and Information Science, Henan Polytechnic University, Jiaozuo, 454000, China; 3 Mathematics and Information Science College, Hebei Normal University, Shijiazhuang, 050024, China

## Abstract

We investigate the distinguishability of orthogonal multipartite entangled states in *d*-qudit system by *restricted local operations and classical communication*. According to these properties, we propose a standard (2, *n*)-threshold quantum secret sharing scheme (called LOCC-QSS scheme), which solves the open question in [Rahaman *et al.*, Phys. Rev. A, 91, 022330 (2015)]. On the other hand, we find that all the existing (*k*, *n*)-threshold LOCC-QSS schemes are imperfect (or “ramp”), i.e., unauthorized groups can obtain some information about the shared secret. Furthermore, we present a (3, 4)-threshold LOCC-QSS scheme which is close to perfect.

Quantum secret sharing (QSS) is an important branch of quantum cryptography, which was simultaneously proposed by Hillery *et al.*^1^ and Cleve *et al.*^2^. It allows a secret to be shared among many participants in such a way that only the authorized groups can reconstruct it. In a (*k*, *n*)-threshold QSS scheme, the dealer distributes a shared secret among *n* participants, and any group of *k* or more participants can collaboratively recover the shared secret, however, no group of fewer than *k* participants can.

During the past two decades, many interesting QSS schemes^1^,^2^,^3^,^4^,^5^,^6^,^7^,^8^,^9^,^10^,^11^ were proposed (for an incomplete list). Recently, Rahaman *et al.* concentrated on the implementation of classical secret sharing by quantum means, and first introduced the theory of local distinguishability of quantum states to the design of QSS scheme^12^. A novel, simple and efficient model of QSS scheme was presented, where the participants only used local quantum operations and classical communication (LOCC), in other words, any joint quantum operations were not required. This QSS model is called LOCC-QSS model. According to the model, a series of (*k*, *n*)-threshold LOCC-QSS schemes were proposed in ref. [12]. The designs of them are based on the local distinguishability of orthogonal multipartite quantum states. That is, some pairs of locally distinguishable orthogonal multipartite entangled states which represent the encoded secret can be collaboratively distinguished by a sufficient number of participants using LOCC, but cannot be distinguished by any fewer than the threshold *k* participants.

The topic of LOCC-QSS is very interesting, meanwhile, it brings us some valuable study points. First, (2, *n*)-threshold LOCC-QSS scheme in ref. [12] is a nonstandard QSS scheme since it needs a strictly restricted condition, i.e., the two cooperating participants must come from two disjoint groups. A natural question how to design a standard (2, *n*)-threshold LOCC-QSS scheme is an open question. Second, all the existing (*k*, *n*)-threshold LOCC-QSS schemes are *ramp* (or “imperfect”) QSS schemes, i.e., there exist some information leakages in these schemes. How to quantify the information leakages and design a (*k*, *n*)-threshold LOCC-QSS scheme of less information leakages or even a *perfect* (*k*, *n*)-threshold LOCC-QSS scheme is also an interesting topic.

In this paper, we revolve around above study points to research and try to solve them. On the one hand, we study the properties of orthogonal multipartite entangled states in *d*-qudit system. What’s more, a standard (2, *n*)-threshold LOCC-QSS scheme is presented, i.e., there is no any restricted condition for the two cooperating participants. On the other hand, we find that all the existing (*k*, *n*)-threshold LOCC-QSS schemes are ramp schemes, i.e., unauthorized groups can obtain some information about the shared secret. Then a near-perfect (3, 4)-threshold LOCC-QSS scheme is proposed.

## Results

### Local distinguishability of quantum states in high dimension system

The paradigm of local distinguishability can be described as follows. Suppose some parties shared a multipartite quantum state which is secretly chosen from a known set of orthogonal quantum states. Their aim is to identify the unknown quantum state perfectly using local operations and classical communication. Numerous interesting results have been reported^13^,^14^,^15^,^16^,^17^,^18^,^19^,^20^,^21^,^22^,^23^,^24^,^25^. Now we discuss the distinguishability about a pair of orthogonal multipartite entangled states by *restricted local operations and classical communication* (rLOCC). Here, rLOCC means only a subset of parties is allowed to communicate with each other^12^.

Let 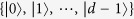 be a standard orthonormal basis of a *d*-dimensional Hilbert space. Consider the following two orthogonal state 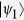, 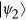, which can act as generalized Bell states in *d*-qudit system.


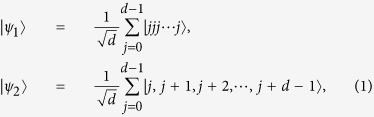


where “+” is performed modulo *d*. For *d* = 2, the two states are known as Bell states. Now we show that the two states in Eq.(1) have the following properties.

Theorem 1. *Two orthogonal entangled states* 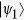, 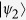 *in Eq.*(1) *can always be exactly distinguished by no less than two cooperating participants using LOCC. But they cannot be distinguished by only one participant.*

*Proof.* On the one hand, according to the forms of the two states, it is easy to obtain a distinguishable protocol. All the cooperating participants (no less than two) measure their own particle in the computational basis 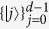 locally. If they have precisely the same results, then the shared state is 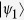. Otherwise, if they have completely different results, the state is 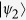.

On the other hand, for the two states, it is straightforward to calculate that any single particle reduced density matrices are *I*/*d*, where *I* is the identity operator in *d*-dimensional system. It means that only one participant cannot obtain any information from his own particle. That is, the two states cannot be distinguished by only one participant.

Now we recall the notion of stabilizer state. The generalized Pauli operators in *d*-dimensional Hilbert space are


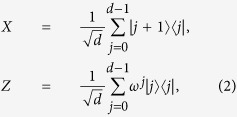


where *ω* = *e*^2*πi*/*d*^. A stabilizer state 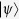 is a state of an *n*-qudit system that is the simultaneous eigenvector, with eigenvalues 1, of a subgroup of *d*^*n*^ commuting elements of the Pauli group which does not contain multiples of the identity other than the identity itself. We call this subgroup as the stabilizer *G* of 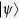
26. When *d* is prime, *G* can always be generated by *n* suitably chosen group elements *g*_*j*_, where the order of each *g*_*j*_ is *d*. 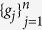 is called a set of generators. When *d* is not prime, one might need more than *n* generators in some cases.

In *d*-qudit system, *d* is a prime. Let us define two sets of quantum operations


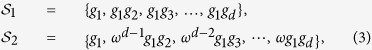


where


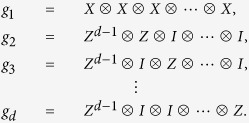


According to the definition of stabilizer, we can easily obtain the following lemma.

Lemma 1. *The elements of* 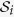 *in Eq.*(3) *constitute the generators set of the stabilizer of the state* 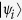, *i* = 1, 2.

It is easy to see that quantum state 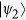 is the unique eigenstate of all the elements of 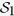 with eigenvalues 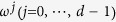. So we have the following theorem.

Theorem 2. *If an unknown state* 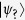 *satisfy:* 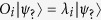, 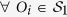
*. Then*

(1) eigenvalues *λ*_*i*_ = 1 if and only if 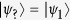, 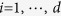;

(2) eigenvalues *λ*_*i*_ = *ω*^*i*−1^ if and only if 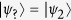, 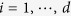.

Note that whether *d* is a prime or not, the two states 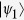, 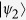 are both the eigenstates of all the elements of 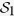. However, Theorem 2 holds only when *d* is a prime. It means that both of the two states can be uniquely determined by the set 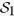 according to eigenvalues. If *d* is not a prime, they may not be uniquely determined by the set 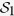 according to eigenvalues.

Theorem 3. *Two orthogonal entangled states in Eq.* (4) *can always be exactly distinguished by no less than three cooperating participants using LOCC. However, they cannot be deterministically distinguished by any two or fewer participants by LOCC.*


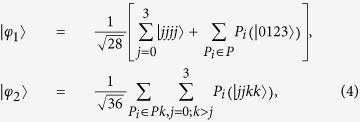


where *P* is the set of all possible distinct permutations.

*Proof.* All the cooperating participants (no less than three) measure their own particle in the computational basis 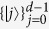 locally. If they have precisely the same results or completely different results, then the shared state is 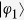. Otherwise, if there exist two participants who have the same results, but their results are different from the other participants’ results, then the shared state is 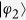.

On the other hand, for the two states, it is straightforward to calculate that any one participant have the same reduced density matrix *I*/*d*, where *I* is the identity operator in *d*-dimensional system. It means that only one participant cannot obtain any information from his own particle. All the bipartite reduced density matrices of 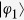 are same because of the symmetry of 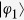, so are that of 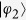. Employing the probability formula of the minimum-error state discrimination 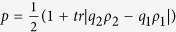
27, where *q*_1_, *q*_2_ are a priori probabilities and *ρ*_1_, *ρ*_2_ are two states, we can calculate that the probability with which any two participants can distinguish the states is 0.5536. It means that two participants cannot perfectly distinguish the two states even if they use joint quantum operations. So the two states cannot be exactly distinguished by any two participants by LOCC. That completes the proof.

### LOCC-QSS

Suppose the sender Alice wants to share a key between *n* separated participants Bob_1_, Bob_2_, …, Bob_*n*_. Only no less than *k* participants can collaboratively recover the shared secret. That is, a (*k*, *n*)-threshold QSS should be designed. Here, we still adopt the basic model of LOCC-QSS in ref. [12] since the basic model is very simple and efficient. For readability, we still use the same notations.

### The standard (2, *n*)-threshold LOCC-QSS scheme

*Step 1*. Alice first prepares a large number (say *L* > *n*) of states chosen randomly from a specified pair of orthogonal *n*-qudit (*n* = *d*) entangled states in Eq.(1) according to her requirement. Let us denote the prepared states by 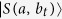 to keep details of each prepared state in each run (run *t* is associated with the prepared state 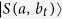 at time *t*). Here, *a* represents the state randomly chosen from a pair of orthogonal states, that Alice prepares at time 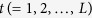, where 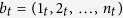 represents the positions of all *n* qudits of a prepared state 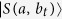 at time *t*, i.e., the position of *i*th qudit of a prepared state *a* at time *t* is denoted by 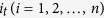.

*Step 2.* Alice prepares at random, a different sequence, 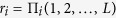 for each Bob_*i*_, and sends the *i*_*t*_th qudit (*i* = 1, 2, …, *n*; *t* = 1, 2, …, *L*) to Bob_*i*_ according to the *r*_*i*_ sequence order, where 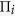 is an arbitrary permutation of the sequence (1, 2, …, *L*). No one has the information about 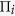 except for Alice. After receiving their associated sequence of qudits, all of the receivers now share *L n*-qudit entangled states 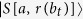. Here 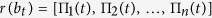.

*Step 3*. Alice now randomly selects some run, say 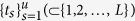, and also computes *n* arbitrarily chosen permutations, *p*_*i*_ of {1, 2, …, *u*}, only known to herself. She then prepares list 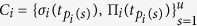 for Bob_*i*_ (for *i* = 1, 2, …, *n*) and sends it to him. After receiving the list *C*_*i*_, Bob_*i*_ measures his 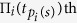 qudit in the 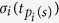 basis and sends the measurement outcome 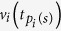 to Alice.

Here Alice choose randomly elements of the set 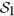 in Eq.(3) to determine Bob_*i*_′s measurement basis. Now we interpret it. First, for the set 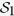, both 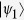 and 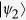 are the eigenstates of the elements of 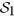. They have the following relation


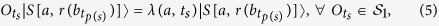


where eigenvalue 
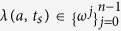
 and 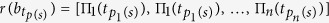. Therefore, all the product of all the local measurement results 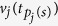 for 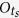 must be equal to the corresponding eigenvalue, i.e., 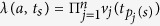. It should be noted that the generalized Pauli operators *X* and *Z* are not Hermite, so *X*, *Z* and 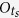 cannot act as observables. However, they are unitary operators. Since the relation between unitary operator *U* and Hermite operator *H* is *U* = exp(*iH*), and they have the same eigenstates. While the above measurement can always be completed using Hermite operator *H* as observables. For simplicity, roughly speaking, one can use the eigenstates of *U* as measurement basis to complete projective measurement, and measurement results can be denoted by eigenvalues.

For example, if Alice chooses 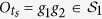, then 
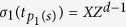
, 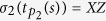, 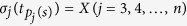. Bob_1_ uses the eigenstates of *XZ*^*d*−1^ as measurement basis to complete projective measurement, and measurement result can be denoted by eigenvalues. Other Bob_*i*_ have the similar way to completed measurement. If the unknown state is 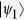, then 
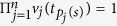
. If the unknown state is 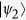, then 
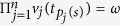
.

In this step, two very important points should be emphasized. First, when Alice prepares list *C*_*i*_ for Bob_*i*_ and sends it to him, Bob_*i*_ still does not know which *n* qudits come from the same entangled state. It is very crucial for design of eavesdropping detection in a concrete LOCC-QSS scheme. Second, Alice starts to send lists *C*_*i*_ only if all of the receivers confirm the receipt of all their *L* qudits.

*Step 4*. For each selected run *t*_*s*_, Alice check whether or not the the product of local measurement results is equal to the corresponding eigenvalue *λ*(*a*, *t*_*s*_), i.e., 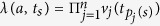. If 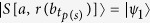, then


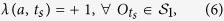


and if 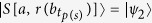, then


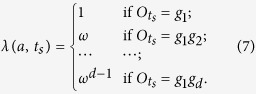


By analyzing the measurement results, Alice can easily detect whether there is an eavesdropper or not. If there is one, she aborts the protocol and starts again from step 1.

*Step 5*. If no eavesdropper is detected, Alice announces, to the respective parties, all qudit positions of an unmeasured state 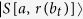. Alice selects this 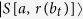 according to her secret *a* (=0 or 1). The mapping between classical bit value and orthogonal entangled states is fixed and is communicated securely from Alice and Bobs in advance. If Alice’s secret is more than one bit, then she reveals the qudit positions of a sequence of unmeasured states 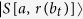.

According to Theorem 2, the states 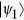, 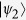 can be uniquely determined by the set *S*_1_ according to eigenvalues if *d* is prime. It makes the protocol be more secure. On the other hand, although 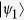 and 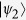 cannot be uniquely determined if *d* is not prime, the protocol is still secure due to the design method of this scheme. It will be shown in the section of security analysis.

Employing Theorem 1, the two states can be exactly distinguished by no less than two cooperating participants using LOCC. But they cannot be distinguished by only one participant. Thus, this is a standard (2, *n*)-threshold LOCC-QSS scheme.

*Example 1.* In a (2,3)-threshold LOCC-QSS scheme, the pair of the states are


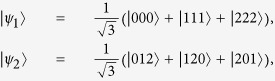


and 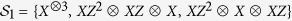. The steps are described in detail in the standard (2, *n*)-threshold LOCC-QSS scheme. Here, we only consider the Step 4. If 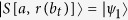, then


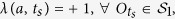


and if 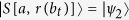, then


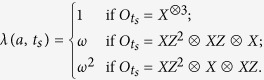


### The security analysis of standard (2, *n*)-threshold LOCC-QSS scheme

The standard (2, *n*)-threshold LOCC-QSS scheme can be regarded as secure because the shared secret cannot be eavesdropped without being detected. Usually, there are three eavesdropping strategies for Eve (she may be dishonest Bob). Now we consider the security of our scheme under the three attacks.

The first eavesdropping strategy is called “intercept-measure-resend”, that is, Eve intercepts the legal particles when Alice sends them to Bob_*i*_, chooses local or global measurement basis to measure them, then resends them to Bob_*i*_. (i) If Eve wants to obtain Alice’s secret, she can choose and measure *n* qudits by global measurement to distinguish the unknown state. However, Eve does not know which *n* qudits come from the same entangled state because Alice has scrambled the order of qudits using permutation 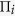, and no one has the information about 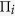 except for Alice. Therefore, this attack will be detected in the eavesdropping detection if Eve chooses this method of attack. (ii) If Eve wants to obtain Bob_*i*_′s secret or wants to obtain Alice’s secret according to more than *t* (threshold value) Bob_*i*_′s secrets, she can measure one or more qudits by local measurement. However, the original correlations of quantum states will be destroyed. For example, Eve chooses computation basis to locally measure the unknown state 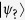. Then 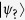 collapses to a product state, which does not satisfy the conditions of eavesdropping detection. This attack will be detected in the eavesdropping detection.

The second one is “intercept-replace-resend”, i.e., Eve intercepts the legal particles and replaces them by her counterfeit ones. If Eve escapes from the detection of Alice, she will obtain Alice’s secret. Now we show that our scheme is security under the attack. (i) If *d* is a prime, according to Theorem 2 Eve cannot find a quantum state which satisfies the conditions of eavesdropping detection to replace the legal particles. (ii) if *d* is not a prime, 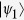, 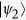 cannot be uniquely determined by the set *S*_1_ according to eigenvalues, that is, there exists another state which satisfies 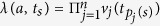. However, Alice has scrambled the order of qudits using permutation 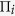, according to Step 2 and 3 anyone does not know which *n* qudits come from the same entangled state except for Alice before the end of the eavesdropping detection. Thus the eavesdropper cannot use the illegal states satisfying 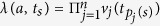 to replace the states which are sent by Alice. Otherwise, the eavesdropping will be found by Alice.

The third one is “entangle-measure”, i.e., Eve entangles an ancilla with the *n*-qudit, at some later time she can measure the ancilla to gain information. Without loss of generality, assume that Eve uses a unitary operator such that the ancilla 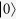 entangles with the quantum state 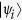, i.e., 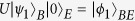, 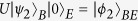, where the subscripts *B* and *E* express the particles belonging to Bob_*i*_ and Eve, respectively. In fact, This kind of attack is general, it contains the above two attacks. Now we will show that the legal particles (*B*) and the ancilla (*E*) must be not entangled if no error is introduced into the QSS procedures. It means that Eve will gain no information about the secret by observing the ancilla.

(i) If *d* is a prime, according to Theorem 2, 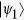 and 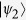 are uniquely determined by *S*_1_. In other words, the state 
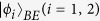
 must be not entangled between *B* and *E*, otherwise, this attack will be detected by Alice with certain probability.

(ii) Next we consider that *d* is not a prime. Firstly, since Eve does not know which *n* qudits come from the same entangled state, the unitary operator can only act on one qudit from 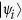 and the ancilla. Secondly, note that the operator *Z*^⊗*n*^ can be generated by the elements of *S*_1_, i.e., 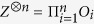, 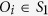 in Eq. (3). Then 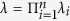, where 
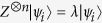
, 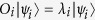. Therefore, only if the state 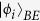 satisfies the property that the product of all Bob_*i*_′s measurement results measured by computation basis is equal to *λ* (=1), may Eve escape from the detection of Alice. So 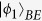 and 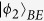 have the form 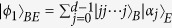 and 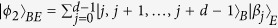, where 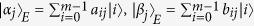. It should be noted that we do not put constraints on the dimensions of 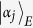 and 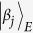. Next we will show that this attack will be detected when participants check eavesdropping with the basis 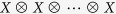. Using the inverse Fourier transform 
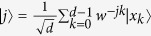
, we consider the form of the *n* legal qudits of quantum state 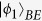 in the Fourier basis, where 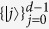 is the computation basis, 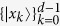 is the Fourier basis and *w* = *e*^2*πi*/*d*^. It is easy to calculate the terms


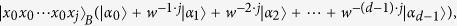


*j* ≠ 0. Obviously, they must be eliminated, i.e., 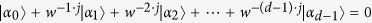, *j* ≠ 0. Otherwise, this attack will be found by Alice. It means 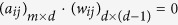, where


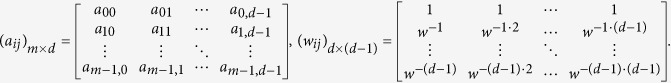


So 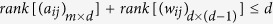. Since the matrix 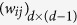 contains a Vandermonde submatrix with the order *d* − 1, we have 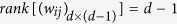. Thus 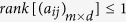. It means that 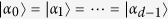 (up to global phase). So 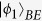 is a product state between legal qudits and the ancilla. The similar discussion can be applied for the analysis of quantum state 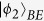, and we can obtain the same result.

Intuitively, maybe it is surprised that the scheme is secure despite 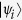 cannot be uniquely determined by the set *S*_1_ for non-prime *d*. The reason is that Alice has scrambled the order of qudits such that the states satisfying conditions of eavesdropping detection are excluded. If the unitary operator can only act on one qudit from 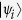 and the ancilla, Eve cannot obtain any information according to the above proof.

### The quantification of information leakages

It is difficult to design a *perfect* (without any information leakage) (*k*, *n*)-threshold LOCC-QSS scheme. At present all of the existing (*k*, *n*)-threshold LOCC-QSS schemes are ramp schemes. We try to quantify the information leakages.

Now we consider conspiracy attack for (*k*, *n*)-threshold LOCC-QSS scheme. If there exist *l*(<*k*) dishonest Bob_*i*_, they can recover the secret together. This attack method is called conspiracy attack. For the (*k*, *n*)-threshold LOCC-QSS scheme^12^, according to the following two intentions, they can choose different ways to eavesdrop.

(i) No matter whether eavesdroppers obtain the shared secret or not, it is not allowed that they obtain a wrong shared secret and disturb the authorized groups to recover the shared secret. For simplicity, the eavesdropping probability of success is called *unambiguous* probability.

(ii) In order to obtain information about the shared secret as much as possible, it is allowed that eavesdroppers minimize the errors that occur in a state discrimination task and can disturb the authorized groups to recover the shared secret. The eavesdropping probability of success is called *guessing* probability.

For the sake of simplicity, we only analyze the example 3 in ref. [12], i.e., (5, 6)-threshold LOCC-QSS scheme. It is easy to be generalized for (*k*, *n*)-threshold LOCC-QSS scheme. First we recall the key steps in the original scheme.

*Step 1*. Alice randomly chooses the states from the pair orthogonal Dicke states


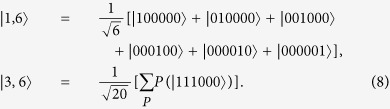


*Step 4*. If 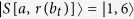, then


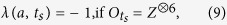


and if 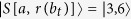, then


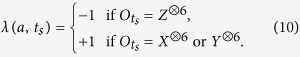


Other steps are similar to the standard (2, *n*)–threshold LOCC-QSS scheme. It should be noted that there is a mistake in original (5, 6)-threshold LOCC-QSS scheme, i.e., if 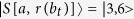, then 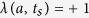, 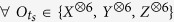.

Now we consider conspiracy attack for (5, 6)-threshold LOCC-QSS scheme.

*The method of conspiracy attack*: these dishonest Bob_*i*_ will faithfully perform the protocol until Alice believes no eavesdropper. For intention (i): when Alice announces all qubit positions of unmeasured states, these *l*(<5) dishonest Bob_*i*_ measure their own qubit in the computational basis locally to recover the secret together. For intention (ii): when Alice announces all qubit positions of unmeasured states, these *l*(<5) dishonest Bob_*i*_ use joint quantum measurement to measure their *l* qubits according to the minimum-error state discrimination.

Now we calculate the probability when *l*(<5) participants recover the secret together.

(1) *l* = 4. For intention (i): if the local measurement results of the four dishonest Bob_*i*_ are three same states 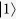 and one state 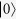 or two states 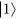 and two states 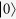, they can determine the state is 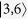. If the local measurement result are four same states 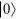, they can determine the state is 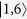. So the *unambiguous* probability is 17/30. On the other hand, for intention (ii), we can calculate that the *guessing* probability is 0.7 according to the probability formula of the minimum-error state discrimination, i.e., the rate of information leakages is 11.87%.

(2) *l* = 3. For intention (i): if the local measurement results of the three dishonest Bob_*i*_ are three same states 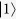 or two states 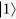 and one state 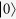, they can determine the state is 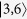. The *unambiguous* probability is 1/4. For intention (ii): the *guessing* probability is 0.625, namely, the rate of information leakages is 4.56%.

(3) *l* = 2. For intention (i): Only the local measurement results of the two dishonest Bob_*i*_ are two same states 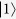, they can determine the state is 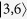. For other local measurement results they cannot distinguish the states. So the *unambiguous* probability is 1/10. For intention (ii): the *guessing* probability is 0.6167, that is, the rate of information leakages 3.97%.

(4) *l* = 1. Obviously, the *unambiguous* probability is zero. The *guessing* probability is 7/12. That is, the rate of information leakages 2.01%.

All the cases can be shown in Table 1, where *l* is the number of dishonest Bob_*i*_, *p*_*u*_, *p*_*g*_, *r* are *unambiguous* probability, *guessing* probability and the rate of information leakages, respectively. The intention (i) is very interesting. Since dishonest Bob_*i*_ can always exactly recover the secret with nonzero probability if the *unambiguous* probability is nonzero, and they cannot disturb the authorized groups to recover the shared secret.

Now we introduce two parameters *k*_1_, *k*_2_ in (*k*, *n*)-threshold LOCC-QSS scheme, denoted as (*k*_1_, *k*_2_, *k*, *n*), to describe the information leakages. It means that (i) any fewer than *k*_1_ participants cannot obtain any information; (ii) any *l* (*k*_1_ ≤ *l* < *k*) participants can obtain the shared secret with *guessing* probability more than 1/2; (iii) any *l* (*k*_2_ ≤ *l* < *k*) participants can obtain the shared secret with nonzero *unambiguous* probability. Obviously, for ramp LOCC-QSS scheme, it has 1 ≤ *k*_1_ ≤ *k*_2_ ≤ *k*. And the more *k*_1_, *k*_2_ are close to *k*, the less information leakages are. For perfect LOCC-QSS scheme, it has *k*_1_ = *k*_2_ = *k*. For the above (5, 6)-threshold LOCC-QSS scheme, it can be denoted as (1, 2, 5, 6)-threshold LOCC-QSS scheme.

Finally, we show that a secure (3, 4)-threshold LOCC-QSS scheme cannot be designed based on the model of (*k*, *n*)-threshold LOCC-QSS scheme in ref. [12]. Since threshold *k* = *n* − *r* + 1 = 3, the distance^12^ *r* between the pair of states is 2. If the pair of states which Alice chooses contains the Dicke state 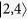, the other is 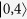, or 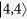. It contradicts with the definition of Dicke state. If the pair of states does not contain the state 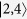, the pair of states must be 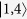 and 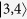. In the stage of eavesdropping detection, only condition 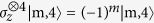 (*m* = 1 or 3) can be used to detect eavesdropping. Obviously it is insecure. Since the eavesdropper Eve can always measure all the qubit in the computational basis then send the post-measurement states to Bob_*i*_, but Alice cannot find Eve’s eavesdropping.

### The (3, 4)-threshold LOCC-QSS scheme

Now we propose a (3, 4)-threshold LOCC-QSS scheme, in which dishonest Bob_*i*_ cannot obtain the shared secret with nonzero *unambiguous* probability. All the steps are similar to the standard (2, *n*)-threshold LOCC-QSS scheme, so we only show the differences.

Step 1. Alice prepares the states, the desired pair of the states are in Eq. (4).

Step 4. If 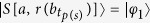, then 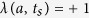, 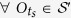, and if 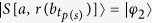, then 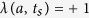, 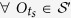, where


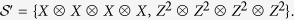


Because both 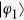 and 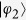 are the eigenstates of the element in 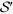 with eigenvalue 1.

According to Theorem 3, we know it is a (3, 4)-threshold LOCC-QSS scheme. Employing the forms of the two states in Eq.(4), it is easy to see that the *unambiguous* probability is zero for any *l* dishonest Bob_*i*_ (*l* < 3). According to the proof of Theorem 3, we know that the *guessing* probability is zero when *l* = 1, and the *guessing* probability is 0.5536 when *l* = 2. The rate of information leakages is 0.83%. So the scheme can be denoted as (2, 3, 3, 4)-threshold LOCC-QSS scheme. It is close to perfect (3, 4)-threshold LOCC-QSS scheme.

## Discussion

In ref. [11], Gheorghiu *et al.* also proposed an efficient QSS scheme by LOCC, which is based on quantum error-correcting codes to distribute a quantum secret. In their QSS scheme, they reduced the required quantum communication at the cost of some classical communication. But our schemes are based on local discrimination of quantum states to distribute classical secrets. And any joint quantum operations and quantum communication are not required in secret recovery stage. Although the designs of these schemes have all used LOCC, their essences are completely different.

In this paper, based on the distinguishability of orthogonal multipartite entangled states by rLOCC in *d*-qudit system, we present a standard (2, *n*)-threshold LOCC-QSS scheme, which work out the open question in ref. [12]. In addition, we take (5, 6)-threshold LOCC-QSS scheme as a example to present that all the existing (*k*, *n*)-threshold LOCC-QSS schemes are ramp schemes. Then we propose a (3, 4)-threshold LOCC-QSS scheme, which is close to perfect. We hope that these results will encourage researchers to study generalized (*k*, *n*)-threshold LOCC-QSS scheme.

## Additional Information

**How to cite this article**: Yang, Y.-H. *et al.* Quantum secret sharing via local operations and classical communication. *Sci. Rep.* **5**, 16967; doi: 10.1038/srep16967 (2015).

## Figures and Tables

**Table 1:** The information leakages of (5, 6) scheme.

*l*	1	2	3	4
*p*_*u*_	0	1/10	1/4	17/30
*p*_*g*_	0.5833	0.6167	0.625	0.7
*r*	2.01%	3.97%	4.56%	11.87%
